# Effectiveness of Adherence Therapy in Adults with Type 2 Diabetes: A Systematic Review

**DOI:** 10.3390/ijerph18094397

**Published:** 2021-04-21

**Authors:** Fatimah Alenazi, Daniel Bressington, Monika Shrestha, Monica Peddle, Richard Gray

**Affiliations:** 1 School of Nursing and Midwifery, La Trobe University, Bundoora 3086, Australia; m.shrestha@latrobe.edu.au (M.S.); m.peddle@latrobe.edu.au (M.P.); R.Gray@latrobe.edu.au (R.G.); 2 Department of Public Health, College of Public Health and Health Informatics, Qassim University, Al Bukayriyah 51941, Saudi Arabia; 3 College of Nursing and Midwifery, Charles Darwin University, Ellengowan Drive, Casuarina 0810, Australia; daniel.bressington@cdu.edu.au

**Keywords:** medication adherence, compliance, type 2 diabetes, diabetes educator, systematic review

## Abstract

Adherence therapy has been shown to be an effective adjunct treatment in long-term conditions including hypertension. The purpose of this study is to review and critically appraise evidence on the effectiveness of adherence therapy as an intervention in adults with type 2 diabetes. A systematic search of clinical trials published between 2005 and January 2020 in databases was undertaken in October 2018 and updated in August 2020. Inclusion criteria were any clinical trials where the population under investigation was adults with type 2 diabetes and the experimental intervention was adherence therapy. Version 2 of the Cochrane risk of bias was used to determine the quality of the included studies. No studies met our inclusion criteria. However, four studies that we excluded at full text screening tested some of the components (e.g., problem solving) of adherence therapy. As is recommended when reporting empty reviews, those studies were synthesized to determine if useful information can be extracted. That no trials of adherence therapy have been reported in type 2 diabetes establishes a potentially important gap in knowledge. This review was registered in PROSPERO (registration number: CRD42019115216) after the initial searches were completed.

## 1. Introduction

According to the World Health Organization (WHO), around 1.6 million deaths were caused by diabetes in 2016 [1]. People with diabetes are generally required to follow a treatment plan, including taking medication every day, following a diet, and exercising regularly. Not taking medication as prescribed may result in health deterioration [2]. There is clear evidence that many people with chronic diseases such as diabetes skip or miss medication doses or ignore treatment altogether [3]. Recommendations by the WHO, the National Institute for Health and Care Excellence (NICE) and the American Diabetes Association (ADA) identify that the patient–provider relationship as playing an important role in diabetes care [3,4,5]. People who feel that providers do not allow enough time to discuss their concerns or treatment options tend not to follow the treatment plan. As diabetes is a long-term chronic disease, adults with diabetes need adequate support to follow guidelines and recommendations. Diabetes care and education specialists have a central role in assisting people who are experiencing difficulty in taking their medication as prescribed [6]. The ADA has made specific recommendations about interventions to facilitate behavior change and improve the overall well-being of people with type 2 diabetes. For example, patient education and support, including social support, medical nutrition therapy, physical activity, smoking cessation, and psychosocial issues, are highly recommended. Furthermore, the ADA stresses the need for the use of patient-centered care to improve overall health status of people with diabetes [7]. Specifically, it promotes the use of the program Diabetes Self-Management Education and Support (DSMES), developed by the Centre for Disease Control and Prevention (CDC), to improve diabetes education and skills including goal setting, problem solving and decision making [7]. DSMES incorporates patient-centered approaches and is intended to be used in daily clinical practice [8]. DSMES is recommended by the ADA; however, a systematic review and meta-analysis of 12 randomized controlled studies reported that although DSMES was effective, the quality of trials underpinning this observation was poor [9]. There is a need for more robust methodological trials before concluding that DSMES is an effective approach in people with type 2 diabetes.

Although the ADA’s recommendations seem comprehensive and practical, they are not tailored to specific clinical issues, such as treatment adherence. For example, the reasons for not taking medication as prescribed are complex and multi-faceted [3]. Therefore, to promote taking medication, healthcare providers must understand the reasons for not taking medication and address them. NICE has published guidelines to assist adults with diabetes in taking their medication as prescribed, which includes four main recommendations: patient involvement in decisions about medicines, supporting and assessing adherence, reviewing medicines, and communication between healthcare professionals [10].

Other guidelines published by the WHO to support adults with diabetes to follow recommended long-term therapies encourage interventions that focus on removing barriers to adherence, as well as addressing adults’ beliefs about their illnesses and treatment [3]. Both guidelines suggest that an ideal solution to improve medication adherence includes following a patient-centered communication style by giving an individualized consultation to involve patients in their health decisions and to address their needs and concerns [3,10].

### 1.1. Adherence Therapy

Many of the recommendations and factors identified to assist adults to take their medication as prescribed are captured in adherence therapy described by Gray et al. [11] Adherence therapy uses a patient-centered approach where the therapist employs motivational interviewing (MI) and cognitive behavioral therapy (CBT) as a set of techniques to support adults to take medication as prescribed [12,13]. In adherence therapy, MI techniques are applied to help patients explore their ambivalence. CBT is a technique used to explore and challenge their beliefs about medication. Adherence therapy is a pragmatic intervention that seeks to use a range of psychological techniques to address factors that are known to impact adherence in people with type 2 diabetes. For example, there is a clear association between beliefs about treatment and adherence [14]; in AT, beliefs are carefully challenged using techniques drawn from CBT—rating the strength of the belief, exploring evidence for and against the belief, highlighting discrepancies between thinking and behavior and then re-rating the belief. The timeline technique is used to help patients reflect on their experiences of living with diabetes and consider lessons learned that can incorporate into a revised plan (e.g., stopping treatment can lead to symptoms getting worse). There are five core techniques in adherence therapy: problem solving, looking back, exploring ambivalence, talking about beliefs, and looking forward. A copy of the adherence therapy manual can be accessed by: https://figshare.com/articles/online_resource/Adherence_therapy_manual/14298335 (accessed on 30 March 2021).

The influence of adherence therapy on improving adults’ ability to follow treatment recommendations has been tested for different diseases, such as hypertension and Parkinson’s. A randomized controlled trial by Alhalaiqa et al. [15] tested adherence therapy in 136 people with hypertension, including adults who were identified as not taking their medication as prescribed. The authors compared participants who received seven sessions of adherence therapy with those who received the usual treatment. There was a significant improvement in taking medication among the adherence therapy group compared to the treatment-as-usual group [15]. Similarly, another randomized controlled trial by Daley et al. [16] involving 76 people with Parkinson’s disease showed an improvement in medication intake and quality of life. Adherence to treatment in Parkinson’s disease is particularly complex as treatment involves long-term treatment with medications that may have long-term negative effects on disease progress—this makes working collaboratively with patients to make shared decisions particularly important. therefore, adherence therapy may be an appropriate intervention for long-term conditions such as Parkinson’s disease and diabetes because there is a strong emphasis on personal control and developing knowledge, skills and attitudes to positively self-manage their medication.

### 1.2. The Need for This Review

A discrete manualized intervention that is based on a sound theoretical underpinning and incorporates the core elements of adherence guidelines by NICE [10] and WHO [3] may be useful as a standardized approach towards managing medication intake in adults with diabetes. As such, adherence therapy is a potential candidate intervention to assist adults with type 2 diabetes in taking their medication as prescribed. Gray et al.’s [11] published approach to adherence therapy includes most of WHO’s and NICE’s guidelines and recommendations, it is based on a sound theoretical base and is manualized. The process and structure of adherence therapy differs from current diabetes interventions. A systematic review investigating the impact of pharmacist-led interventions in improving adherence in people with type 2 diabetes identified 59 trials where more than 75% of the trials were educational interventions [17]. Another systematic review examining the effectiveness of adherence intervention in people with type 2 diabetes by Williams et al. [18] showed that 19 out of 27 trials were fundamentally designed as educational interventions, and most of them were delivered in a community-based approach. A Cochrane review investigating the effectiveness of adherence intervention indicated that there is no intervention for improving adherence in people with type 2 diabetes [19]. In systematic reviews of adherence interventions [20], discrete interventions have been shown to be potentially effective, but these have not been combined into a comprehensive long-term approach. Furthermore, it might be argued that enhancing treatment adherence is part of diabetes self-management [21]. However, the emphasis in self-management packages around medication adherence seems to be largely focused on education, and, overall, the quality of evidence showing that self-management is effective is debatable, while the quality of trials has been reported as having a potential risk of bias [9]. Therefore, adherence therapy could be beneficial for adults with type 2 diabetes. Additionally, the fact that adherence therapy is a manualized intervention makes it possible to be included as routine practice for diabetes care and education specialists.

Before conducting a randomized controlled trial testing the adherence therapy intervention, it is important to understand the current state of evidence regarding the intervention and its effects on medication intake in adults with type 2 diabetes. To the best of our knowledge, no systematic reviews have focused on using adherence therapy to improve the clinical outcomes of adults with type 2 diabetes.

### 1.3. Objectives

This systematic review aims to identify, select, critically evaluate and synthesize findings from any clinical trial that reports on the effectiveness of using adherence therapy to improve glycated hemoglobin (HbA1C)—the average level of blood sugar in the past two to three months [22]—A1C and/or medication intake in adults with type 2 diabetes.

## 2. Methods

### 2.1. Protocol and Registrations

This review followed the Preferred Reporting Items for Systematic Reviews and Meta-Analyses (PRISMA) reporting guideline [23]. The protocol was retrospectively (after initial searches were undertaken) registered with the PROSPERO registry on the 7 January 2019 (Registration number: CRD42019115216).

### 2.2. Eligibility Criteria

Studies were included in the review if: participants were adults aged 18 years of age or over diagnosed with type 2 diabetes (of any duration), any clinical trial design (single group trial, randomized control trial, control clinical trial, pilot or feasibility studies) was used, the intervention was described as adherence therapy, participants were of any gender, and the manuscript was written in the English language.

### 2.3. Information Sources

Searches were undertaken on the 11 October 2018 (and updated on the 30 August 2020) in the following databases: MEDLINE, EMBASE, EMCARE, CINHAL, CENTRAL, and PsycINFO for studies published after 2005 (when adherence therapy was first reported Gray et al. [11]. The authors did not search gray literature (e.g., unpublished reports or manuscripts) because a replicable search strategy cannot be described, studies have often not been peer reviewed, and publications may not be a permanent record [24,25]. The authors searched unpublished trials by manually checking the following clinical trial registries: www.clinicaltrials.gov (accessed on 20 April 2021), Australian New Zealand Clinical Trial Registry (ANZCTR), and Current Controlled Trials (ISRCTN).

### 2.4. Search

Our search strategy was developed in collaboration with an information scientist but was not externally peer reviewed by a second librarian. Our initial search strategy was developed in MEDLINE using the medical subject headings (MeSH terms), and keywords and was subsequently adapted for five other databases: (‘Diabetes mellitus type 2′ OR ‘type 2 diabetes mellitus’ OR ‘type 2 diabet*’ OR ‘diabet* type 2′ OR ‘Diabetes mellitus type two’ OR ‘type two diabetes mellitus’ OR ‘type two diabet*’ OR ‘diabet* type two’).tw. OR exp Diabetes Mellitus, Type 2/AND ‘adherence therapy’.mp. OR exp Medication Adherence/or exp Patient Compliance/.

### 2.5. Study Selection

Relevant studies from databases were imported into EndNote X9 software. Studies were then uploaded to the Covidence website (https://www.covidence.org/home accessed on 11 October 2018) where duplicates were removed. Covidence is a systematic review management tool that enable multiple researchers to work on a review at the same time in a fully auditable way. Two independent reviewers (FA and MS) completed title and abstract screening against the review inclusion criteria. Conflicts were resolved by a third reviewer (AA). Full texts were then uploaded into Covidence by FA. Full-text review—against inclusion criteria—was again undertaken by two reviewers (FA and MS) with discrepancies resolved by a third (AA).

### 2.6. Data Collection Process

Two researchers (FA and MS) completed data extraction in Covidence. Any discrepancies were resolved by a third reviewer (AA).

### 2.7. Data Items

The following data were extracted from the included studies: author, country, clinical setting, study design, comparator intervention, delivery mode (e.g., individual, telephone, group), total number of sessions, sample size, mean (SD) post treatment A1C scores, measure of taking medication as prescribed, and mean (SD) post treatment adherence scores.

### 2.8. Risk of Bias in Individual Studies

All studies meeting the inclusion criteria were to undergo quality appraisal. Risk of bias for randomized controlled trials was to be determined using the Cochrane risk of bias tool-version 2 (performance, detection, attrition, reporting, other) for each type of study [26]. For the non-randomized controlled trail study, the Cochrane Risk of Bias in Non-randomized Studies—of Interventions (ROBINS-I) assessment tool were to be used [27].

### 2.9. Summary Measures

The principal summary measure was difference in post treatment means (A1C and taking medication as prescribed).

### 2.10. Synthesis of Results

Synthesis of findings aimed to combine the principal results of the studies included in the review to present a summary of the current evidence. If two or more studies reported on the efficacy of adherence therapy using A1C, a meta-analysis was to be employed to present an aggregate synthesis [27]. For studies reporting different outcome measures (e.g., taking medication as prescribed, beliefs about treatment), a narrative synthesis was to be used to summarize findings.

### 2.11. Publication Bias

A funnel plot generated using REVMAN was to be used to determine if there is any potential for publication bias [28].

## 3. Results

### 3.1. Study Selection

**Figure 1:**
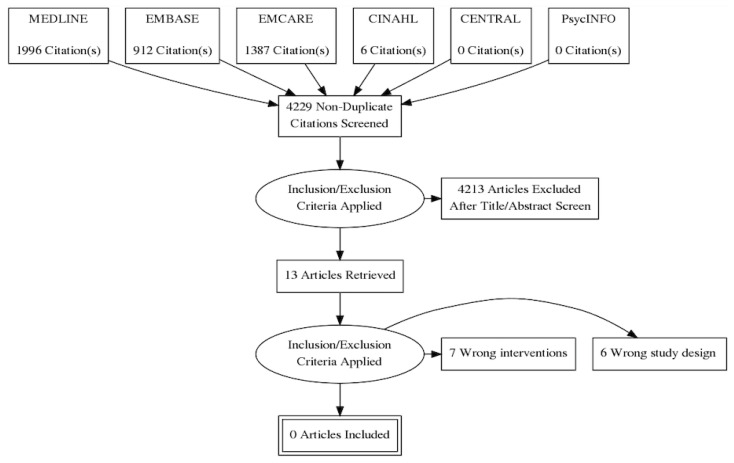
PRISMA flow diagram.

Figure 1 shows the flow of studies through the review. The database search retrieved 4300 papers. No trials were identified by searching trial registries. After screening the title and abstract of papers, 13 studies which met the initial inclusion criteria were included in a full text review. During the full text review, no studies met the inclusion criteria. Six studies were excluded because of the study design (non-interventional) and seven were excluded as they did not specifically test adherence therapy as an intervention. The authors are therefore reporting an empty review where no studies met pre-defined inclusion criteria. Lang et al. [29] recommends that empty reviews should consider evidence from trials excluded at full text screening if they were relevant to the review question, even if they did not explicitly meet review inclusion criteria.

Of the seven studies excluded at full text screening, three were not relevant to this review because they did not test any aspect of adherence therapy (Brunton, [30] Lim et al. [31] Manju et al. [32]). Brunton [30] reported an RCT testing the effectiveness of GLP-1R agonists in diabetes. Lim et al. [31] and Manju et al. [32] tested pharmacist-delivered didactic patient education. Adherence therapy does have an educational component; however, the focus of adherence therapy is on exchanging information (finding out from the patient what information the patient needs and then providing this information in a factual way) that is discrete from the didactic model tested in the Lim et al. [31] and Manju’s [32] trials.

Four studies excluded at full-text screening were relevant to the review question, providing evidence about the effectiveness of discrete components (e.g., motivational interviewing techniques and reviewing medication histories) of the adherence therapy intervention. The reviewers provided data extraction, quality appraisal and a narrative synthesis of these four trials: Adikusuma and Qiyaam [33], Bindu Murali et al. [34], Erku et al. [35], and Fall et al. [36].

### 3.2. Study Characteristics

The characteristics of the four relevant studies are shown in Table 1. Two studies were randomized controlled trials (Erku et al. [35] and Fall et al. [36]), one a controlled clinical trial (Adikusuma and Qiyaam [33]), and the final paper by Bindu Murali et al. [34] reported on a before and after study. All of the studies recruited and delivered experimental interventions to participants in a hospital setting; however, the authors did not specify if these were inpatient or outpatient settings. The intervention was delivered by a pharmacist in three studies [33,34,35]; however, it was not reported who delivered the intervention in the fourth study [36]. Interventions were tested in four different countries: Indonesia [33], India [34], Ethiopia [35], and France [36]. The total number of participants was 351, of which 198 received a novel adherence intervention. In the three controlled trials, 94 participants received a novel adherence and 133 received a control intervention; an active control was used in one trial [36], and treatment as usual was used in the other two studies [33,35]. The duration of treatment was only reported in one trial [33], with the duration of treatment in other trials not clearly detailed.

#### 3.2.1. Outcomes Measures

All four trials reported that participants improved in taking medication as prescribed. Two studies measured improving medication intake as prescribed using the Morisky Medication Adherence Scale [34,35], one using the Medication Adherence Questionnaire [36], and one using pill count (reported as a percentage of prescribed medication) [33]. One study had A1C as an outcome [33].

#### 3.2.2. Components of Adherence Therapy Tested

The included trials tested a number of component interventions that approximate those used in adherence therapy, e.g., looking back, problem solving, talking about beliefs, and looking forward. Adikusuma and Qiyaam [33] and Erku et al. [35] both tested problem-solving issues with medication and discussed with participants their beliefs about treatment. However, it was not clear from the manuscript the procedures or processes the authors followed when delivering these interventions. In the Bindu Murali et al. [34] and Fall et al. [36] studies, participants were asked to look back and reflect on their experiences of taking medication. Again, precisely how this was done is not described in detail in the manuscripts. Asking participants to consider how medication might enable them to achieve life goals (approximating the looking forward intervention in Adherence Therapy) was tested in the Bindu Murali et al. [34] trial, although precise details of the intervention are sparse in the paper.

### 3.3. Results of Individual Studies

All of the four included studies reported that there was a statistically significant improvement in taking medication as prescribed. None of the trials reported effect sizes in the manuscript. Improvements in A1C were reported in the single trial where this was measured as an outcome.

No harms or adverse events were reported in any of the four studies.

### 3.4. Risk of Bias within Studies

The risk of bias for the two randomized and two non-randomized trials is shown in Table 2 and Table 3, respectively. Both randomized trials were rated as having a high risk of bias across all domains with the exception of selection of participants. Non-randomized trials were also rated as having a high risk of bias across all domains. None of the four included trials reported a trial registration number.

## 4. Discussion

This is the first systematic review to examine the effectiveness of adherence therapy in improving glycemic control in people with type 2 diabetes. No studies were identified specifically testing Adherence Therapy in adults with type 2 diabetes, and therefore an empty review is reported. An empty review is defined as a review that does not include any studies based on their inclusion criteria [29]. There is considerable debate about the contributions of empty reviews to evidence-based practice. On the one hand, reviews with no included studies are a valuable source of evidence since they highlight the knowledge gaps and can be helpful for policymakers/researchers to decide to investigate the topic further [37,38,39]. On the other hand, researchers argue that empty reviews may be too narrow and offer a false conclusion about the state of knowledge because the research used strict and restrictive inclusion criteria. Therefore, an empty review might suggest to policymakers that no evidence exists on a particular topic when in fact it does [38]. It could be argued that prior to undertaking a full systematic review, researchers should first undertake a scoping review of the literature that would capture all relevant studies, not just randomized controlled trials.

Currently, there are no guidelines that set out a clear way of reporting the findings and conclusions of an empty review. However, it is well known that the purpose of systematic reviews is to summarize the findings of the research question [40]. If an empty review answers the research question without creating confusion in the reader by clearly stating that the findings of excluded reverent studies were not searched systematically, then the review is considered to be useful as it provides information for further research [41].

### 4.1. Summary of Evidence

The American Association of Diabetes Educators (AADE) has defined the diabetes care and education specialist as a “healthcare professional who have experience in the care of people with diabetes and have achieved a core body of knowledge and skills in the biological and social sciences, communication, counseling, and education” [42]. Therefore, diabetes care and education specialists are required to be able to assess patient’s willingness and readiness in taking medication, design and implement intervention based on recent standard and published guidelines.

Studies included in this review were complex, using more than one component—education, motivation, SMS reminders, and reviewing medication—which makes it hard to specify which components were the most effective. From the full text reviewing stage, the authors provided assessment of the four studies that tested many components of adherence therapy (e.g., problem solving, talking about beliefs about medication). Yet, it is important to consider that adherence therapy components were applied with other non-adherence therapy components such as didactic educational sessions.

Additionally, the four relevant studies were rated as having a high risk of bias and were not clearly reported, negatively impacting upon replicability. Studies lacked detailed descriptions of the intervention components, which led to unclear conclusions. For example, Erku et al. [35] described the intervention as in-person sessions, although some were conducted telephonically “whenever necessary”. The conditions regarding the telephone sessions and the number of sessions were not specified. Furthermore, the definitions of non-adherent patients were not provided in the inclusion criteria. Although a significant improvement was reported in all of the outcome measures in the included studies, the effect size was not reported, and calculation was not possible due to a lack of information.

The outcomes of this review indicate that based on the current evidence, it may be possible to improve taking medication as prescribed; however, there is no specific intervention that is effective for all persons [10]. Evaluating interventions that do not consider guidelines and recommendations to improve taking medication as prescribed might lead to inadequate knowledge and evidence. Investigators should provide rationales for specific intervention components reported in the literature, relative to the underpinning reasons that they address adults not taking medication as prescribed to justify including them in the intervention. The components of research and the methods used in interventions that aim to improve taking medication as prescribed must be enhanced to be able to draw a clear conclusion.

### 4.2. Implications for Regulators

AT is a comprehensive candidate adherence intervention that has not been tested in people with type 2 diabetes. There is a clear gap in knowledge about how to address the prevalent problem of poor treatment and self-management adherence. Policy makers should temper their advice to clinicians based on this gap in knowledge.

### 4.3. Future Research Agenda

AT is an intervention that has shown promise in other long-term conditions, the approach is novel because it combines core elements from MI, CBT, psychoeducation and offers a patient-centered approach based on a careful and considered adherence assessment. There is a good case to make for undertaking an appropriately powered clinical trial in people with type 2 diabetes.

### 4.4. Limitations

The aim was to review all of adherence therapy interventions applied for adults with type 2 diabetes. Not including qualitative papers, grey literature and limiting the review to English articles only is a limitation of our review, as some important data might be missed.

## 5. Conclusions

The findings of this study provide support for the need for a standardized intervention that aims to improve medication intake and treatment plans in adults with type 2 diabetes. Adherence therapy is a potentially beneficial intervention in improving medication intake as prescribed and captures most of the WHO and NICE recommendations for such interventions. However, this review found that adherence therapy has not been used in this group. Despite this, some elements of adherence therapy have been tested in other studies involving adults with type 2 diabetes. Overall, the study findings suggest that future trials of manualized discrete interventions that aim to improve taking medication as prescribed in adults with type 2 diabetes (perhaps including adherence therapy) are warranted in population. Furthermore, this study highlights the importance of studies adopting a well-controlled design to minimize bias and demonstrate efficacy and for published manuscripts to ensure all information is clearly reported to strengthen the replicability of the intervention. This empty review may justify performing a comprehensive scoping review to identify studies that have tested the individual elements of the adherence therapy intervention.

## Figures and Tables

**Table 1:** Characteristics of the relevant studies.

Citation	Country	Clinical Setting	Study Design	Intervention Group	Comparator Group	Delivery Mode	Number of Sessions	Sample Size	Mean (SD) Post Treatment A1C Scores (Intervention, Comparator)	Taking Medication as Prescribed Measurement: Mean (SD) Post Treatment Scores (Intervention, Comparator)
Adikusuma and Qiyaam, (2018) [28]	Indonesia	Hospital	Controlled clinical trial	Pharmacist led	Treatment as usual	Counselling and text messages about medication reminders and motivation	Two counselling sessions and 15 SMS	40 (20 pharmacist group, 20 comparator group)	Intervention group (6.97 ± 0.67), comparator group (8.03 ± 0.89)	Intervention group (11.33 ± 8.47), comparator group (2.18 ± 15.56)
Erku et al. (2017) [30]	Ethiopia	Hospital	Randomized controlled trial	Pharmacist led using medication therapy management	Treatment as usual	Face to face counselling and telephone calls as needed	3 visits (phone call as needed)	127 (62 pharmacist group, 65 comparator group)	Not reported	Not reported
Fall et al. (2013) [31]	France	Hospital	Randomized controlled trial	Not reported	Control group (recall negative emotions) and (recall positive emotions)	-	Not reported	80 (20 mastery perceptions intervention group, 20 threat perceptions intervention group, 20 nondiabetic positive emotions comparator group, 20 nondiabetic negative emotions comparator group)	Not reported	Medication Adherence Questionnaire (4.88 ± 0.17)
Bindu Murali et al. (2016) [29]	India	Hospital	Prospective interventional study (pre- and post-test)	Pharmacist led using medication therapy management	No comparator	Face to face counselling	Not reported	104 intervention group	Not reported	Not reported

**Table 2:** Risk of bias in randomized controlled trials (Cochrane Risk of Bias Version 2).

Citation	Bias Domains						Overall Risk of Bias Rating
	Random Sequence Generation	Allocation Concealment	Blinding of Participants	Blinding of Outcome	Incomplete Outcome Data	Selective Reporting	
Erku et al. (2017) [30]	Some concern	Some concern	Some concern	Some concern	Some concern	Some concern	High risk
Fall et al. (2013) [31]	Some concern	Some concern	Some concern	Some concern	Low risk	Some concern	High risk

**Table 3:** Risk of bias in non-randomized trials (ROBINS-I).

Citation	Bias Domains							Overall Bias
	Confounding	Selection of Participants	Classification of Interventions	Deviations from Intended Interventions	Missing Data	Measurement of Outcomes	Selection of the Reported Result	
Adikusuma and Qiyaam, (2018) [28]	Serious	Low	Low	Low	Low	Moderate	Low	Serious
Bindu Murali et al. (2016) [29]	Serious	Low	Moderate	Low	NI	Low	Low	Serious

## Data Availability

Data sharing not applicable.
